# Evaluation of three methods for delineation and attenuation estimation of the sinus region in MR-based attenuation correction for brain PET-MR imaging

**DOI:** 10.1186/s12880-022-00770-0

**Published:** 2022-03-17

**Authors:** Jani Lindén, Jarmo Teuho, Mika Teräs, Riku Klén

**Affiliations:** 1 Turku PET Centre, University of Turku and Turku University Hospital, Kiinamyllynkatu 4-8, 20521 Turku, Finland; 2 Department of Mathematics and Statistics, University of Turku, Vesilinnantie 5, 20014 Turku, Finland; 3 Department of Medical Physics, Turku University Hospital, Hämeentie 11, 20521 Turku, Finland; 4 Institute of Biomedicine, University of Turku, Kiinamyllynkatu 10, 20014 Turku, Finland

**Keywords:** MRAC, CTAC, PET-MRI, Sinus region, Attenuation correction, TOF

## Abstract

**Background:**

Attenuation correction is crucial in quantitative positron emission tomography-magnetic resonance (PET-MRI) imaging. We evaluated three methods to improve the segmentation and modelling of the attenuation coefficients in the nasal sinus region. Two methods (cuboid and template method) included a MRI-CT conversion model for assigning the attenuation coefficients in the nasal sinus region, whereas one used fixed attenuation coefficient assignment (bulk method).

**Methods:**

The study population consisted of data of 10 subjects which had undergone PET-CT and PET-MRI. PET images were reconstructed with and without time-of-flight (TOF) using CT-based attenuation correction (CTAC) as reference. Comparison was done visually, using DICE coefficients, correlation, analyzing attenuation coefficients, and quantitative analysis of PET and bias atlas images.

**Results:**

The median DICE coefficients were 0.824, 0.853, 0.849 for the bulk, cuboid and template method, respectively. The median attenuation coefficients were 0.0841 cm^−1^, 0.0876 cm^−1^, 0.0861 cm^−1^ and 0.0852 cm^−1^, for CTAC, bulk, cuboid and template method, respectively. The cuboid and template methods showed error of less than 2.5% in attenuation coefficients. An increased correlation to CTAC was shown with the cuboid and template methods. In the regional analysis, improvement in at least 49% and 80% of VOI was seen with non-TOF and TOF imaging. All methods showed errors less than 2.5% in non-TOF and less than 2% in TOF reconstructions.

**Conclusions:**

We evaluated two proof-of-concept methods for improving quantitative accuracy in PET/MRI imaging and showed that bias can be further reduced by inclusion of TOF. Largest improvements were seen in the regions of olfactory bulb, Heschl's gyri, lingual gyrus and cerebellar vermis. However, the overall effect of inclusion of the sinus region as separate class in MRAC to PET quantification in the brain was considered modest.

**Supplementary Information:**

The online version contains supplementary material available at 10.1186/s12880-022-00770-0.

## Background

Attenuation correction (AC) of the bone is of paramount importance for the quantitative accuracy of positron emission tomography (PET), especially in the brain. Transmission-based attenuation correction (TXAC) with a rotating ^68^Ge rod source can be considered as a “gold standard” method for attenuation correction, allowing to measure the attenuation coefficients directly. Whereas, computed tomography (CT)-based attenuation correction (CTAC) could be considered as a “silver standard” [1], where bilinear scaling method of CT Hounsfield Units (HU) to attenuation correction factors is used to derive an attenuation map (µ-map).

Contrary to TXAC or CTAC, magnetic resonance based attenuation correction (MRAC) does not allow a direct measurement of electron density information. This is because magnetic resonance (MR) images represent the tissue proton densities and tissue relaxation values, which cannot be directly translated to electron density information. For accurate attenuation correction on PET-MRI systems, several methods have been introduced, where the advanced methods have achieved an accuracy of ± 5% in quantitative accuracy of PET when compared to CTAC [2]. These methods can be roughly divided into atlas-based, emission-based and segmentation-based methods. These methodologies and the physical basis of attenuation correction have been discussed in review articles [3–6].

To shortly summarize the main methods for MRAC, atlas and template-based methods allow to calculate a pseudo-CT, or a synthetic-CT image, which can be created by, for example, co-registering a single CT or a database of CT images to the individual anatomy of a subject presented by the MR image [7–9]. Recently, several methods based on machine learning have also been introduced [10]. Emission-based methods use raw PET data to mathematically reconstruct the attenuation sinogram for MRAC [4, 11–13]. Furthermore, on time-of-flight (TOF) compatible PET-MRI systems, incorporating TOF data in the image reconstruction might bring further increases in quantitative accuracy [14, 15].

In addition to these approaches, the MR images can be segmented into different tissue classes. When using conventional T1/T2-weighted MRI sequences, the accurate delineation of bone becomes challenging, as bone has a similar T2* relaxation time as air. However, with advanced segmentation methods, such as probabilistic atlas-based segmentation [16], bone can be segmented also with conventional MRI sequences. In addition, ultra-short echo time (UTE) sequences and zero echo time (ZTE) sequences allow to visualize and segment bone directly from MR images [17, 18]. Advanced segmentation-based methods have shown very good accuracy when compared to emission- and atlas-based methods [2]. However, there are a few remaining challenges in segmentation-based MRAC, which still need to be addressed.

One of the remaining challenges is to accurately segment and assign attenuation coefficients to regions such as the nasal sinuses, which contain a mixture of bone, air and soft tissue [19]. Manual contouring of the nasal sinus region has been suggested [20, 21], but preferably, the subject-specific mixtures of different tissues should be accounted on an individual basis in an automated way. Previously, regional masks and templates have been suggested to improve the segmentation accuracy in these regions [22–24]. In addition, inclusion of TOF in image reconstruction may further reduce inaccuracies in these regions [14]. Thus, it is important to study alternative approaches to accurately delineate and to implement methods to accurately estimate the attenuation coefficients in this region. Furthermore, the accuracy of the methodology should be investigated with and without TOF.

For the basis of evaluation in this paper, we used a segmentation-based MRAC method based on Statistical Parametric Mapping software (SPM8) introduced in [25, 26]. The method was modified to use the segmentation engine in SPM12 and included three different methods for tissue delineation in the sinus region. Two new methods were developed to provide a proof of concept for accounting the subject-specific variation of the attenuation coefficients in the sinus region. The method accuracy across the brain were evaluated with and without TOF reconstruction, in regard to the activity recovery in PET.

## Materials and methods

### Clinical subject data

The data consisted of 10 subjects (three males, seven females), suspected of memory disorders, which had undergone a PET-CT and PET-MRI scan, were used in this retrospective study. The subjects had undergone both PET-CT and PET-MRI during the same imaging session (seven subjects) or PET-CT and PET-MRI performed on separate days (9 days, 6 months 19 days and 7 months 3 days apart). The mean and standard deviation of subject age, weight, and injected dose of [^18^F]-FDG were: 53 ± 14 years, 73 ± 19 kg, and 277 ± 47 MBq. The mean ± standard deviations of the scan start times were 80 ± 20 min after the injection for the group which had undergone imaging during subsequent sessions. All PET-CT and PET-MRI acquisitions were performed using the standard protocol for neurological imaging.

The study was conducted as a retrospective registry study at Turku PET Centre (study number: T7/2021). The need to acquire an active informed consent from the individuals included in the study was waived and the study protocol was approved by Turku University Hospital Research Board and the legislative team. The requirements for ethical review in Finland are stipulated primarily in the Medical Research Act (488/1999, as amended) and the Act of the Medical Use of Human Organs, Tissues and Cells (101/2001 as amended). Ethical review is statutorily required for interventional medical research and some circumstances for studies on human organs, tissues or cells. According to Finnish legislation, no ethical assessment or approval by an independent review board is mandatory for registry studies. The study was conducted in accordance with the Declaration of Helsinki.

### PET-MRI system

The MRI and PET acquisitions were performed sequentially with the Philips Ingenuity TF PET-MRI (Philips Healthcare, Cleveland, OH, USA). Ingenuity TF is a combination of a PET subsystem (Gemini TF with TOF capabilities) and an MRI system (Achieva 3 T X-series). The PET system has 4 mm × 4 mm × 22 mm lutetium oxyorthosilicate crystals arranged in 28 detector modules. The PET system has a 180 mm axial field of view (FOV) and a coincidence window of 6 ns with an energy window of 460–665 keV. The maximum gradient strength of the MRI system is 40 mT/M and the slew rate is 200 T/m/s. The performance of the system is evaluated in more detail in [27].

### MRI and PET image acquisition

Anatomical MRI (atMR) from the vendor-based MRAC acquisition was used to derive all MRI-based μ-maps. The atMR is a T1-weighted 3-D fast field echo (FFE) sequence with 2 mm isotropic voxel size. The acquisition parameters were: echo time (TE) of 2.16 ms, FOV 320 × 320 mm^2^, flip angle (FA) of 10°, repetition time (TR) of 4.18 ms, and an acquisition time of 84 s. The geometry correction on the MRI system was applied with the option value set as "default”. The SENSE 8-channel head coil was used for all PET-MRI imaging. For PET, a 15-min acquisition was performed in list-mode over one bed position covering the head region with transaxial acquisition FOV of 256 × 256 mm^2^.

### CT and PET image acquisition

For reference measurement for attenuation correction, CTAC data from the PET-CT examination was used. The Discovery 690 PET-CT (General Electric Healthcare, Milwaukee, WI, USA) was used to perform the CTAC acquisitions. The CT system is a LightSpeed volume computed tomography with 64 slices. All CTAC maps were acquired with a low-dose CT acquisition protocol using a tube voltage of 120 kV and 10 mAs with dose modulation. The physical performance of the PET system can be found in [28].

### MR image segmentation and tissue classification

The method in this paper is based on the one described in [25, 26, 29]. The method uses tissue probability maps from SPM8 to create MR-based attenuation maps. The probability maps are segmented from T1-weighted MR images by use of the New Segment function in SPM8 (Wellcome Trust Centre for Neuroimaging, University College London, UK).

In this paper, we implemented the following modifications in the original method. First, the method was updated to use the Segment function in SPM12. As SPM12 introduces two new parameters for post-processing of the tissue probability maps and alters one parameter, adjustment of the segmentation settings in SPM12 was needed for optimal bone delineation. The final parameters used for the Segment function in SPM12 were very light (0.0001) for bias regularization (2, 2, 2, 3, 4, 2) for number of gaussians, 0 for strength of the Markov random field (MRF) cleanup, no cleanup, (0, 0.001, 0.5, 0.05, 0.2) for warping regularization and 0 mm for the smoothness parameter. These parameters were then used to segment the T1-weighted MR images to grey matter, white matter, cerebrospinal fluid, scalp, skull, and air.

Thereafter, separate binary masks for each tissue class were created, which were then combined and given discrete attenuation coefficients. The threshold values for segmenting tissues were set to: 0.25 for air, 0.50 for soft tissue, 0.25 for bone, and 0.5 for brain tissues. The final attenuation map consisted of four tissue classes with attenuation coefficients of 0.0 cm^−1^ for air, 0.096 cm^−1^ for soft tissue, 0.985 cm^−1^ for brain tissue, and 0.151 cm^−1^ for bone, as specified in [25, 30]. All of the processing is performed in an automated MATLAB 2018b pipeline to derive MRI-based μ-maps for PET-MR image reconstruction.

### Image processing and modelling workflow for the sinus cavity

As there is no separate class for the sinus region in the Segment function of SPM12, it is mostly classified as bone. However, this region is anatomically a mix of air, soft tissue and fine bone. To take this challenging region into account, we modified the image processing pipeline to include three alternative methods for sinus delineation. The first method is called the bulk method and it was implemented directly as described in [29].

In short, the bulk method is based on registration of a CT template, to create a specific mask in the sinus region to assign a fixed attenuation coefficient of 0.100 cm^−1^ for that region. The drawback of the method is that it needs an additional registration of a CT-based template to delineate the sinuses, assuming that the subject has normal anatomy. Another drawback is the use of a bulk assignment of an attenuation coefficient.

Two new methods presented in this study are called the cuboid method and the template method. The cuboid method is based on matching a cuboid-shaped mask to the individual anatomy of the subject. In the template method, the cuboid is inverse transformed from a template in Montreal Neurological Institute (MNI) space to subject anatomy by using the vector fields from SPM12 Segment function. Finally, the voxels within the cuboids are converted into air and soft tissue using a MRI-CT conversion model. The processing pipelines are described in detail below.

### Processing pipeline for the cuboid method

First, an initial binary mask of the air inside the sinus cavity is created by intersection of air and soft tissue masks. The initial mask includes the throat, large air cavities in the sinus, and voxels that do not match with anatomical locations of air cavities. To delineate the air cavities only, the mask was summed across all slices, and the slice sums were made into a line plot. An initial search for the largest air cavities was performed using the largest local maximum preceding the largest drop in the inside air as a criteria for finding the optimal slice. Air cavities in this slice were then chosen by a center of mass method. Thereafter, a region-growing algorithm was applied to delineate all air cavities. The entire air cavity segmentation process is described in detail in Appendix A. After air delineation, a cuboid is fitted to the resulting mask to delineate the region applied for the conversion model.

To get an anatomically fitting cuboid, a set of cuboids are fitted around the air cavities. A detailed description of this method can be found in Appendix B. A large cuboid is placed initially in the front half of the head extending above and below the air cavities. Then sub-cuboids within the maximum cuboid are fitted and evaluated. The best cuboid is a tradeoff between the amount of bone segment covered, and the size of the cuboid, where the final cuboid is selected based on the following criteria:

1 $$CG = \frac{{\mathop \sum \nolimits_{i \in C} p_{i} }}{{\left( \frac{c}{4} \right)^{2} }},$$

where *CG* is the value of the cuboid goodness, *C* is the cuboid being tested, *c* is the perimeter of the cuboid and *p*_*i*_ is the bone segment probability value for the voxel *i*. In this way, the more voxels with considerable bone probability there are within the cuboid, the higher its goodness *CG* will be. The sub-cuboid with the largest CG is then selected as basis for further processing.

### Processing pipeline for the template method

An alternative method for delineating the sinus cavities was evaluated based on matching a cuboid template to an individual anatomy by inverse transformation. This method is based on a cuboid template in MNI space, similarly to the regional masks used in [23]. The inverse transformation fields from the Segment function in SPM12 are used to transform the cuboid template back to the individual space of the subject. The resulting mask may no longer strictly be a square cuboid since the subjects’ heads can be slightly tilted and morphed in various directions. The inverse transformed cuboid is then used as a basis for further processing.

### MRI-CT conversion model for the sinus region

**Fig. 1:**
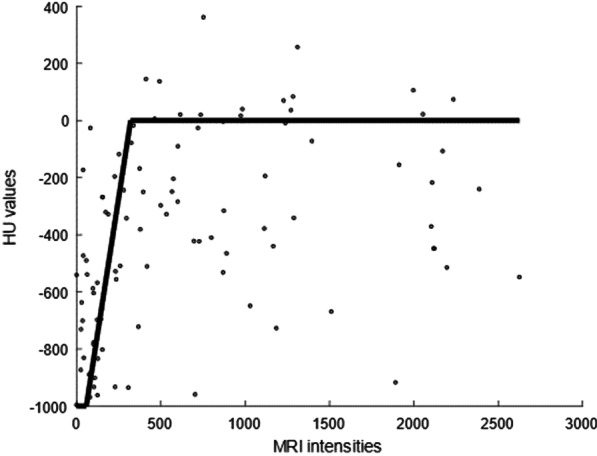
An example of a conversion curve with MRI intensities (x-axis) and corresponding CT HU values (y-axis) for one subject. The average of all the sampled conversion curves from all subjects used as the final model for MRI-CT conversion

Once the cuboid location was fixed for either of the methods, all bone segment voxels were converted to air, soft tissue, or a mix of both using an MRI-CT conversion model. The model was derived based on the 10 subjects as follows. The data scatter plot (Fig. 1; Additional file 1: Fig. S2) of the MRI and CT values in the bone segment voxels within the cuboid masks showed that there are concentration points around − 1000 and 0 HU values on a wide range of MRI values and a smaller amount of points scattered around these two lines. Thus, a three-step conversion model was implemented which followed a horizontal line at − 1000 HU (corresponding to air), a diagonal line from − 1000 to 0 HU (corresponding to mix of different tissues), and a horizontal line at 0 HU (corresponding to soft tissue).

The start and end points from − 1000 to 0 HU were estimated from the data. By using randomly selected 100 voxels in the MRI images from an sinus mask of one subject, possible combinations for MRI-CT values were fitted, where a square sum of the shortest distance between the MRI value at − 1000 HU and MRI value at 0 HU was used for optimization criteria. This randomization and fitting process was repeated 100 times for each subject, effectively creating a bootstrap estimate. For the final MRI-CT conversion model, the average over all 100 randomized estimates for MRI values at − 1000 HU and 0 HU were selected as final conversion points.

### PET image reconstruction

Three sets of MRI-based attenuation maps were created for each subject. The only difference between the maps was the method used to delineate the sinus region. CTAC data was used as the reference method for attenuation correction. CTAC data was converted from HU to linear attenuation coefficients using the bilinear transform described in [31]. In cases where the attenuation map in CTAC did not cover the entire head of the subject, the attenuation map was complemented by soft tissue from MRAC.

To ensure accurate anatomical alignment, all CTAC and MRAC images were realigned and co-registered to non-attenuation corrected PET images. Co-registration was performed using rigid image registration with normalized mutual information as implemented in SPM12. The images were then resliced to the same voxel dimensions as used for the clinical MRAC. All images were smoothed into PET resolution of 5 mm to match the PET-MRI intrinsic resolution.

Thereafter, the MRAC and CTAC images were imported with the raw PET data to the PET-MRI reconstruction system and PET images were reconstructed using TOF and non-TOF reconstruction algorithms. For non-TOF reconstruction, a LOR-RAMLA with two iterations and 33 subsets, matrix size of 128 × 128 × 90 and isotropic voxel size of 2 mm was used. The reconstruction parameters used were alpha = 6.3716, radius = 2.8, blob increment = 2.0375, and relaxation parameter = 0.035. For TOF reconstruction, a TOF-BLOB-OSEM algorithm with 3 iterations and 33 subsets, matrix size of 128 × 128x90 and a voxel size of 2 mm was applied. The reconstruction parameters used were alpha = 4.1338, radius = 2.3, blob increment = 2.0375 and relaxation parameter = 1.0.

All reconstructions included the necessary corrections for image quantification, including random events, scatter, dead time, decay, and normalization. The head coil template and patient table were inserted automatically by the reconstruction software.

### MR-based attenuation map and PET evaluation

#### DICE analysis

DICE coefficients were calculated from the sinus region and from the entire image stack. The sinus region was defined as slices ranging from 50 to 70, which corresponds to 4.2 cm in size. Estimating the DICE coefficients over the sinus region allows to estimate bone delineation accuracy in that region. Whereas, measurement over image volume is indicative of the accuracy in skull bone delineation. DICE coefficients for CTAC (*A*) and MRAC (*B*) were calculated in Eq. 2:

2 $$DICE = \frac{{2 \left| {(A \cap B)} \right|}}{\left| A \right| + \left| B \right|},$$

where $\left| X \right|$ denotes the number of voxels in the binary mask *X*. Voxels where *HU* > 157 were included in the masks.

An HU threshold of 157 HU was used throughout DICE analysis, which corresponds to attenuation coefficient of 0.105 cm^−1^, which is higher than soft tissue, but slightly lower than the reported attenuation coefficient for spongy bone at 0.11 cm^−1^ [32]. This threshold value was selected as it has been used previously as a boundary value between bone and non-bone tissue [33]. We report the change in the DICE coefficients as percentages between cuboid and template methods compared to the bulk method. To indicate the general trend, median change is also presented.

### Correlation analysis

Correlation analysis was performed to investigate the accuracy of the skull bone delineation with each of the methods. Correlation data was sampled from each subject separately. Again, voxels with HU value > 157 were included in the analysis. Each subject was sampled 100 times for 200 random voxel pairs. Due to the sample size, Pearson correlation was used. These correlations were then plotted in a boxplot for each method to evaluate the distribution of the values.

### Attenuation coefficient analysis

The average attenuation coefficient in the sinus region was calculated for all attenuation maps. For the analysis, a volume of interest (VOI) consisting of the nasal sinuses was delineated manually over CTAC in Carimas 2.9 (Turku PET Centre, Turku, Finland). The same VOI was then applied on the three MRAC methods.

A leave-one-out method to test out-of-sample accuracy of the MRI-CT conversion model was performed. In the leave-one-out validation, data for a single subject was removed and the conversion model was calculated for the remaining nine subjects. The resulting model would then be used to perform MRI-CT conversion for the removed subject. This process was then repeated for all subjects.

### VOI analysis of PET images

In quantitative evaluation of reconstructed PET images, automatic VOI analysis was employed using a well-established anatomical atlas provided in automated anatomical labeling software [34], using in total 35 anatomical VOI from the grey matter. Individualization of the atlas was based on the spatial mapping from the MNI space to individual space using the vector fields extracted by the Segment function in SPM12. The atlas image was masked in the individual space with a grey matter mask with a lower threshold of 0.8.

In the analysis, we calculated the relative difference of the total mean activity in each VOI between CTAC and MRAC reconstructed PET images. The mean relative difference was calculated for each VOI using Eq. 3:

3 $$VOIDiff_{rel} = \frac{PETMRAC - PETCTAC}{{PETCTAC}},$$

where PETMRAC denotes the VOI activity measured from MRAC reconstructed PET with different MRI-based μ-maps, while PETCTAC denotes the VOI activity measured from CTAC reconstructed PET. We report the median of the average relative differences over all subjects per VOI.

### Visual evaluation of PET Atlas and bias Atlas images

Atlas PET images representing the mean and standard deviation of PET uptake (kBq/mL) across the subject group were calculated with all MRAC methods and CTAC. In the evaluation of global bias distribution, mean bias atlas images were calculated as described by [25, 35]. All atlas images were masked with a mask covering the entire brain of the subject.

## Results

### Visual inspection

**Fig. 2:**
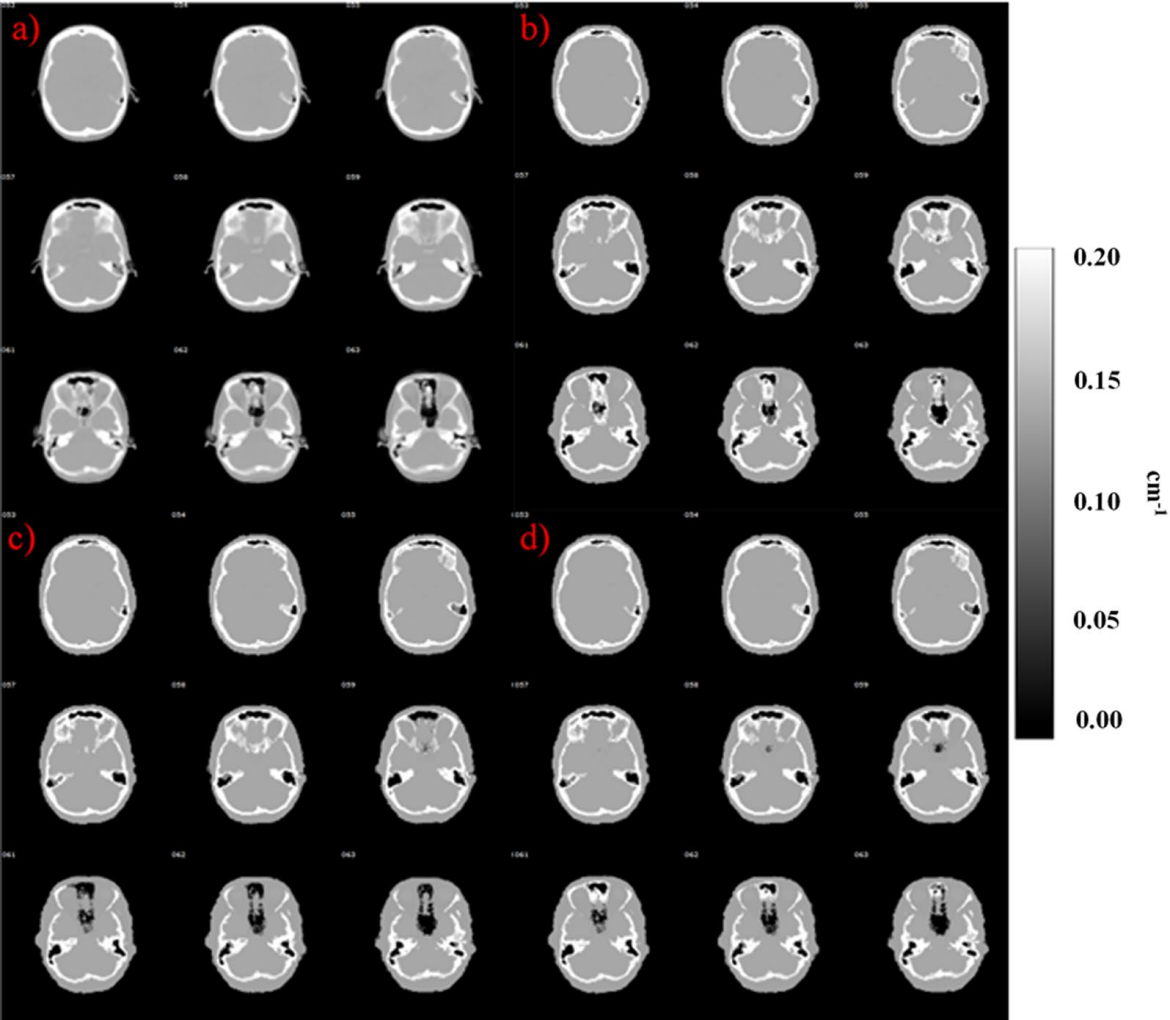
Comparison of attenuation maps using **a** CTAC, **b** bulk, **c** cuboid and **d** template method from subject 10. In this subject, the cuboid provided the best delineation of the air cavities compared to CTAC

Figure 2 shows a visual inspection of CTAC and MRAC images. The air pathways and regions with mixture of tissue are better delineated with cuboid and template methods. Of note is that these regions are often mapped as bone in the bulk method, resulting in overestimation of attenuation.

**Fig. 3:**
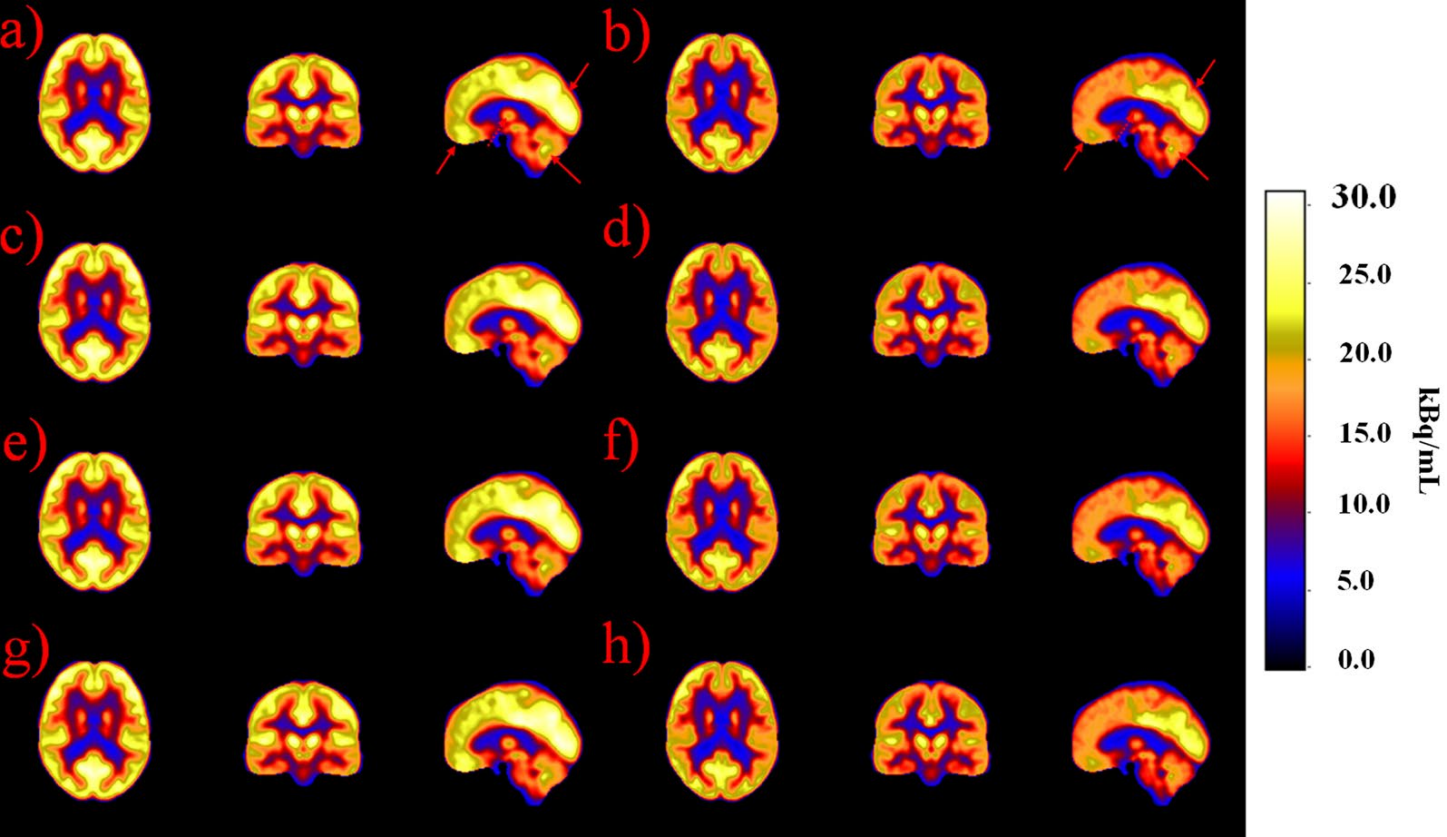
Atlas images representing mean PET images with non-TOF and TOF reconstructions. Non-TOF reconstructions are represented with: **a** CTAC, **c** bulk, **e** cuboid and **g** template method. TOF reconstructions are represented with **b** CTAC, **d** bulk, **f** cuboid and **h** template method. Very slight differences between the methods can be noted in the region of cerebellum, occipital lobe, frontal lobe and in the mid-brain. These are highlighted with red arrows in subfigures **a** and **b**

**Fig. 4:**
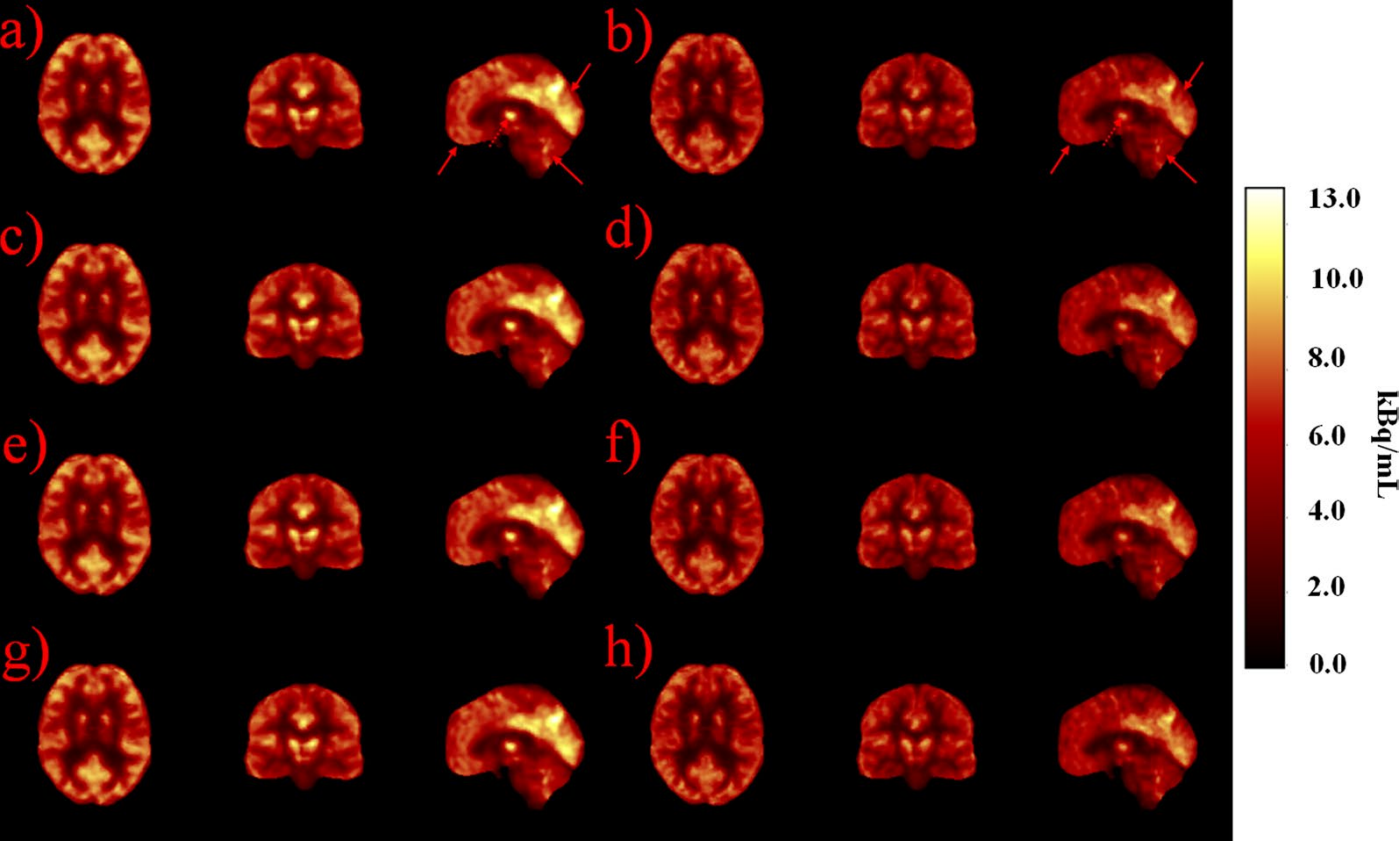
Atlas images representing standard deviation PET images with non-TOF and TOF reconstructions. Non-TOF reconstructions are represented with: **a** CTAC, **c** bulk, **e** cuboid and **g** template method. TOF reconstructions are represented with **b** CTAC, **d** bulk, **f** cuboid and **h** template method. Very slight differences between the methods can be noted in the region of cerebellum, occipital lobe, frontal lobe and in the mid-brain. These are highlighted with red arrows in subfigures **a** and **b**

Figures 3 and 4 show the mean and standard deviation PET atlas images using CTAC and different MRAC methods. The PET uptake values are very similar between the methods. Very slight differences can be noted visually in the cerebellum, in the mid-brain region and in the regions of the occipital and parietal lobes in both the mean and standard deviation images.

### DICE analysis

The DICE values from the sinus region are in Table 1. The DICE coefficients increased with the cuboid and template methods, although the total changes were small, given the size of the region. The DICE values in the entire image were in the similar range with average values of 0.846 ± 0.038 (mean ± standard deviation) for the bulk, 0.847 ± 0.042 for cuboid and 0.845 ± 0.041 and template method.

**Table 1:** DICE coefficients between different methods for slices 50–70 with precentage difference to the reference

Subject number	Bulk	Cuboid	Template	Difference cuboid—bulk (%)	Difference template—bulk (%)
1	0.845	0.852	0.856	0.88	1.37
2	0.826	0.853	0.853	3.26	3.24
3	0.843	0.872	0.872	3.43	3.49
4	0.725	0.771	0.768	6.32	5.99
5	0.820	0.856	0.846	4.47	3.26
6	0.737	0.748	0.751	1.49	1.79
7	0.751	0.771	0.766	2.74	2.05
8	0.868	0.882	0.882	1.59	1.57
9	0.823	0.859	0.853	4.31	3.56
10	0.825	0.832	0.829	0.92	0.52
Median	0.824	0.853	0.849	3.00	2.65

### Correlation analysis

**Fig. 5:**
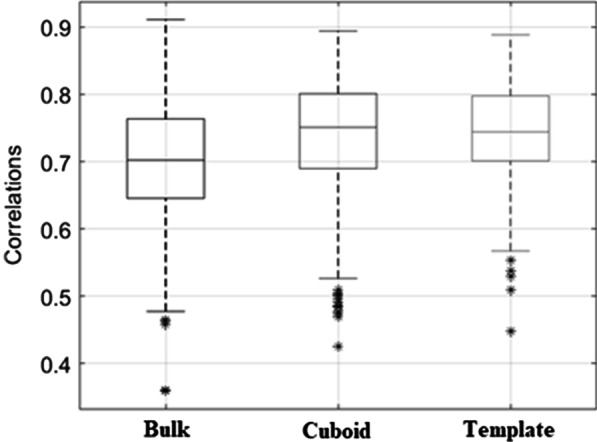
Correlations between CTAC and MRAC images. The data points in the box plots are correlations between all sampled points in a subject. Asterisks (*) are outlier observations

Figure 5 shows the correlation between CTAC and MRAC with all methods. Increased correlation between CTAC and MRAC with the cuboid and template methods were seen.

### Attenuation coefficient analysis

The attenuation coefficients from the sinus region using leave-one-out validation with CTAC and MRAC are in Table 2. Attenuation coefficients in the bulk method are systematically higher than other methods and CTAC, indicating overestimation.

**Table 2:** Average attenuation coefficients (cm^−1^) measured from the sinus region using leave-one-out validation

Subject number	CTAC	Bulk method	Cuboid method	Template method
1	0.0841	0.0893	0.0856	0.0865
2	0.0888	0.0916	0.0867	0.0886
3	0.0929	0.0945	0.0928	0.0931
4	0.0781	0.0849	0.0824	0.0824
5	0.0944	0.0970	0.0948	0.0948
6	0.0761	0.0827	0.0799	0.0798
7	0.0840	0.0859	0.0865	0.0833
8	0.0769	0.0842	0.0801	0.0839
9	0.0861	0.0899	0.0875	0.0892
10	0.0759	0.0811	0.0776	0.0794
Median	0.0840	0.0876	0.0860	0.0852

The values for cuboid and template methods have been converted using leave-one-out validation

**Fig. 6:**
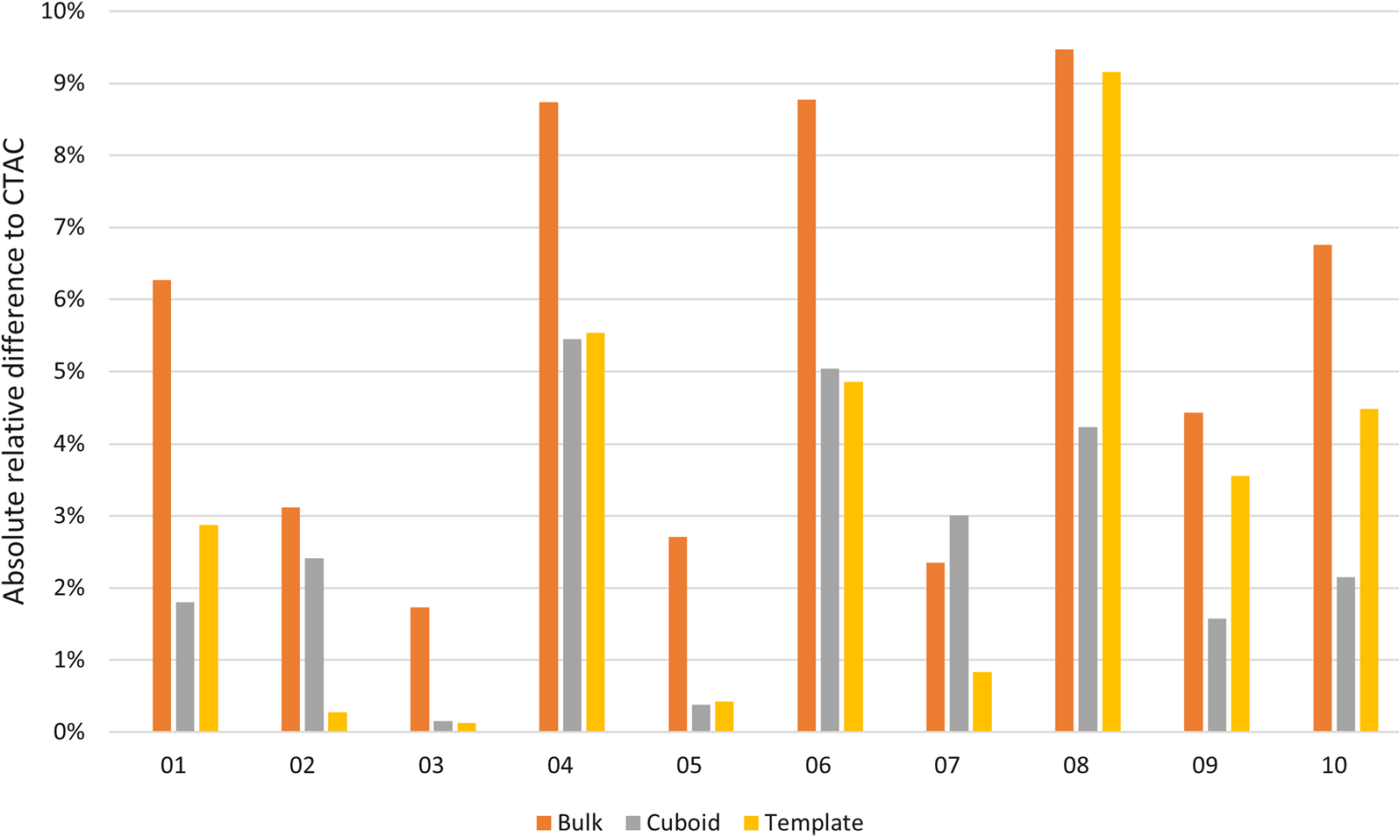
Absolute relative difference of the attenuation coefficients between CTAC and MRAC in the sinus region per subject. The values for the cuboid and template methods are calculated using leave-one-out method. Smaller numbers indicate a better match between MRAC and CTAC

The absolute relative difference of the attenuation coefficients between CTAC and MRAC from all leave-one-out models are plotted in Fig. 6. The cuboid and template methods can reach near parity with the CTAC, whereas the bulk method again overestimates the attenuation coefficients. The difference in attenuation coefficients between the methods is 5% at maximum within subjects.

### PET image analysis

#### VOI analysis

The median errors (Eq. 3) from the VOI level analysis are given in Table 3. The proposed methods show improvement over the bulk method for both non-TOF and TOF reconstruction, although the changes are small. For non-TOF reconstruction, 17 of VOIs (49%) show improvement for both the cuboid and template method. For TOF reconstruction, both methods show higher improvement of 28 VOIs (80%). Of note is that the difference between the methods is smaller when using TOF.

**Table 3:** Median errors for different methods between MRAC and CTAC

	NON TOF reconstruction				TOF reconstruction			
	Bulk (%)	Cuboid (%)	Template (%)	Improvement (%)	Bulk (%)	Cuboid (%)	Template (%)	Improvement (%)
Precentral	− 0.13	− 0.22	− 0.24	0.10	0.16	0.06	0.08	− 0.09
Rolandic_Oper	0.54	0.40	0.38	− 0.15	0.75	0.63	0.64	− 0.12
Supp_Motor Area	0.20	0.14	0.15	− 0.06	0.22	0.14	0.16	− 0.07
Olfactory	**0.36**	**0.11**	**0.10**	− **0.26**	0.49	0.32	0.34	− 0.16
Region #5	− *0.12*	− *0.34*	− *0.39*	*0.24*	0.24	0.09	0.08	− 0.15
Frontal_Sup	− 1.21	− 1.28	− 1.31	0.09	− 0.26	− 0.28	− 0.30	0.03
Frontal_Mied	− 0.60	− 0.64	− 0.65	0.05	− 0.14	− 0.17	− 0.16	0.03
Frontal_Inf	0.49	0.32	0.28	− 0.19	0.60	0.58	0.57	− 0.02
Rectus	**2.32**	**1.70**	**1.88**	− **0.53**	1.30	1.23	1.19	− 0.09
Insula	1.27	1.10	1.16	− 0.14	1.53	1.48	1.46	− 0.06
Cingulum_Ant	1.06	1.02	1.00	− 0.06	1.20	1.04	1.04	− 0.16
Cingulum_Mid	0.92	0.85	0.85	− 0.06	0.72	0.65	0.65	− 0.07
Cingulum_Post	0.44	0.29	0.27	− 0.16	1.17	0.91	0.85	− 0.29
Hippocampus/ ParaHippocampal	0.11	− 0.15	− 0.19	0.07	**0.90**	**0.34**	**0.27**	− **0.60**
Amygdala	0.11	− 0.02	− 0.03	− 0.09	0.77	0.41	0.34	− 0.39
Calcarine	− 0.20	− 0.27	− 0.27	0.07	0.83	0.52	0.49	− 0.33
Cuneus	− 0.54	− 0.62	− 0.61	0.07	0.17	− 0.01	0.02	− 0.15
Lingual	− 0.28	− 0.37	− 0.38	0.09	**0.71**	**0.09**	**0.11**	− **0.60**
Occipital	− 1.16	− 1.22	− 1.23	0.07	− *0.49*	− *0.72*	− *0.70*	*0.22*
Fusiform	− *0.86*	− *1.07*	− *1.08*	*0.22*	− *0.05*	− *0.73*	− *0.77*	*0.71*
Postcentral	0.16	0.11	0.12	− 0.05	0.34	0.21	0.23	− 0.12
SupraMarginal	**0.25**	**0.05**	**0.05**	− **0.20**	**0.73**	**0.30**	**0.34**	− **0.41**
Angular	0.73	0.65	0.65	− 0.08	0.56	0.42	0.45	− 0.12
Precuneus	− 0.25	− 0.31	− 0.33	0.07	0.33	0.22	0.20	− 0.12
Paracentral Lobule	− 0.29	− 0.34	− 0.33	0.05	0.18	− 0.05	− 0.02	− 0.14
Caudate	− 0.05	− 0.17	− 0.16	0.12	0.48	0.25	0.29	− 0.21
Putamen	1.25	1.22	1.22	− 0.03	1.54	1.35	1.34	− 0.20
Pallidum	0.80	0.73	0.77	− 0.05	0.86	0.70	0.69	− 0.16
Thalamus	− 0.25	− 0.46	− 0.43	0.19	0.28	− 0.16	− 0.11	− 0.14
Heschl	**0.26**	**0.04**	**0.07**	− **0.20**	0.74	0.34	0.36	− 0.39
Parietal	− 0.23	− 0.43	− 0.42	0.20	0.40	− 0.07	− 0.03	− 0.35
Temporal	− 0.40	− 0.47	− 0.45	0.06	− 0.39	− 0.52	− 0.50	0.12
Vermis	0.37	0.22	0.18	− 0.17	**0.98**	**0.23**	**0.20**	− **0.77**
Cerebellum Crus	− *0.50*	− *1.59*	− *1.37*	*0.98*	− *0.40*	− *1.91*	− *1.92*	*1.52*
Cerebellum	− *0.73*	− *1.59*	− *1.36*	*0.74*	− *0.36*	− *1.83*	− *1.37*	*1.24*
Error minimum	− 1.21	− 1.59	− 1.37		− 0.49	− 1.91	− 1.92	
Error maximum	2.32	1.70	1.88		1.54	1.48	1.46	

The cuboid and template methods provide at least equal or better performance than the bulk method, although cerebellum notably worsens in both TOF and non-TOF reconstruction. This effect is caused by the overestimation of attenuation in the bulk method. Improvement denotes the average change from bulk to cuboid and template methods. Four largest and smallest improvements are noted in bold and italics respectively

The magnitude of the error in the VOI analysis was low, where majority of the regions show errors of less than 2%. The largest bias measured was in the gyrys rectus with the non-TOF reconstructions with 2.32% with the bulk method, reduced to below 2% with the cuboid and template methods. With TOF reconstructions, all regions produced a bias of less than 2%, with largest errors in the putamen (1.54%) and insula (1.53%). The cuboid and template methods had increased error in the cerebellum region from 1% to 1.5%.

### Bias Atlas images

**Fig. 7:**
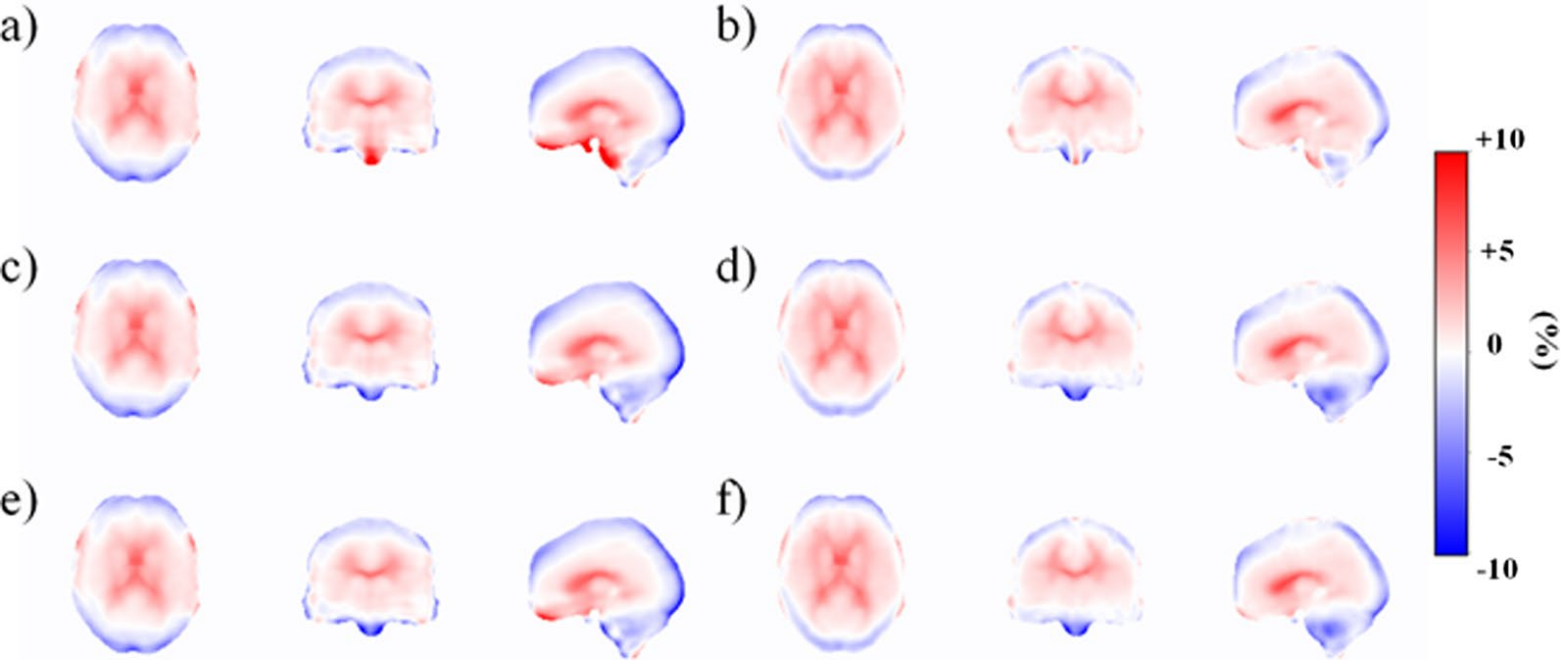
Bias atlas images showing the voxel-by-voxel relative difference between MRAC and CTAC based PET images in non-TOF and TOF reconstruction. Non-TOF reconstructions are represented with: **a** bulk, **c** cuboid and **e** template method. TOF reconstructions are represented with **b** bulk, **d** cuboid and **f** template method. Local overestimations are seen with the bulk method whereas overestimations are reduced with the proposed methods and with TOF

The atlas images showing the bias distribution across the brain are shown in Fig. 7. The bias distribution follows the findings from the VOI analysis, where the cuboid and template methods show improved accuracy in the brain regions near the sinus area. The bulk method shows overestimations in the regions near the sinus area and regions near the ventricles. The bias is distributed more uniformly in the cerebellum area with the cuboid and template methods, whereas there are both over- and underestimations with the bulk method.

The TOF reconstructions using the same attenuation maps show a similar trend with a slightly lower magnitude in absolute bias level. The overestimations seen in the regions near the sinus cavities are also reduced in the TOF reconstructed images. Performance of the cuboid and template methods is improved, resulting to reduction of bias near the sinus region, near the skull and in the cerebellum.

## Discussion

We introduced two new methods as a proof-of-concept for accurate delineation and modelling of the attenuation coefficients in the nasal sinus region and assessed their accuracy in a combined PET-MRI system. In addition, we investigated if TOF improves the regional accuracy in different anatomical regions of the brain using the proposed methods.

There are some minor differences between the cuboid and the template method. The cuboid method is more computationally demanding, as it tries to fit an optimal cuboid into each subject’s individual anatomy using the bone probability map. Fitting might not be optimal should the MR images of a subject be severely deformed, as no tilting is implemented in the fitting of the cuboid due to computational demands and fit time. This could be addressed by modifying the search algorithm by adding tilts.

The template method circumvents this by using each subject’s deformation fields which allow the cuboid to be translated to the individual anatomy. However, if the subject’s anatomy has details that do not translate properly into MNI space, this can cause problems, as a normal anatomy is assumed. Also, the template method does not use the bone probability maps to aid the cuboid fitting and thus it’s accuracy is dependent on the quality of the template registration.

Of note is that none of the subjects showed worse performance with the two new methods compared to the bulk method, in majority of the brain regions, especially after inclusion of TOF information. The results also show improvement in terms of bone delineation accuracy, accuracy of the modeled attenuation coefficients, and improvement of quantitative accuracy in PET with the proposed methods. Finally, the results indicate that the cuboid method has a slight performance edge over the template method.

### Visual comparison

Local improvements were seen using the cuboid and template method over the bulk method in MRAC images (Fig. 2). The PET image analysis showed very minor changes when inspecting the images visually (Figs. 3 and 4). TOF showed a reduced standard deviation in all of the proposed methods.

### DICE and correlation analysis

The cuboid and template methods had increased DICE values in the nasal sinus region by 3% (Table 1) with higher correlations (Fig. 5) compared to the bulk method. The effect is small but consistent. As the sinus region is only a small part of the image and the skull is modeled equally in all methods, it is expected that the differences are regionally concentrated. The whole image DICE values are also of the same magnitude, indicating that the skull delineation accuracy is not affected by the new methods.

In comparison, using SPM12 for processing of the tissue probability maps resulted in increased DICE values in the skull region 0.85 ± 0.04, compared to 0.76 ± 0.05 reported previously using SPM8 [25]. Thus, using SPM12-based MRAC pipeline also improved the bone delineation accuracy.

### Attenuation coefficient analysis

The cuboid and template methods had the smallest error compared to CTAC in the sinus area (Table 2; Fig. 6). The bulk method had the worst performance in nine out of 10 subjects. The error for the bulk method is 5.4% on average compared to an average error of 2.6% and 3.2% on the cuboid and template method. The cuboid method had slightly better performance compared in four subjects and equal performance in four subjects. This would indicate that accounting for individual anatomy in the cuboid method allows us to delineate the region better than using a template.

### VOI analysis

In general, the level of error in regional analysis was very small, with errors less than 2.5% in non-TOF reconstructions and less than 2% in TOF reconstructions (Table 3). However, some regional differences could be noted. The method performance was improved slightly from the previous [25], and is in the similar range as the recent state-of-the-art MRAC methods [6]. Currently, the accuracy of our method is close to the method in [36], where the authors reported an average error of 3.31%.

The error in the Cerebellum increased with the cuboid and template methods with approximately 1% to 1.5% in non-TOF and TOF reconstructions (Table 3). This is caused by two factors. First, the bulk method overestimates attenuation in the sinus region. Second, this overestimation results in both over- and underestimations in the cerebellum region which cancel each other out in VOI analysis, producing a lower bias compared to the proposed methods.

There is some variability between the methods and non-TOF and TOF reconstructions on which brain regions improve the most. The best performing regions were olfactory bulb, Heschl's gyri, lingual gyrus, and cerebellar vermis, while cerebellum and cerebellum crus performed generally worst. Thus, these regions benefit the most by the inclusion of separate class of the nasal sinus region and addition of TOF in reconstruction.

Previous studies have reported an improvement on quantification accuracy in the cerebellum region when the sinus region was accounted in the attenuation maps [19], which is in contrary to our study. However, the method in [19] implements continuous attenuation coefficients for the skull bone, resulting in reduction of bias in the cerebellum which contains typically very dense bone [23], whereas our method implements a single value for the skull bone.

### Bias Atlas images

The non-TOF bias atlas images show improvement with the cuboid and template methods over the bulk method (Fig. 7). The bulk method shows both over- and underestimations in the region of the cerebellum. In addition, the regions near the sinuses are overestimated. With the cuboid and template methods, overestimation is reduced in the ventral and medial parts of the brain, regions near the sinuses, and the error distribution in the cerebellum is more uniform, although it is underestimated compared to the bulk method. This is again due to the bulk method causing local over- and underestimations due to systematically higher attenuation coefficients.

In comparison to a recent study [37], which detected an anterior–posterior located bias in the brain, the bias in our study is deviating more from the mid-brain (positive bias) to the cortical regions (negative or zero bias), given the differences in MRAC approaches in these studies. In addition, TOF seems to reduce the error in the PET images similarly to [38], regardless of the AC method. The methods actually perform much closer to each other as the imperfections in the attenuation maps are better accounted with TOF, even with the limited time resolution (525 ps) of our PET-MRI system.

In summary, our CTAC-MRAC and PET analysis confirms the hypothesis of Sousa et al., who suspected that the effect of attenuation coefficient assignment in the sinus region is modest [37], especially after inclusion of TOF. However, our methodology provides alternative approaches to be applied for more accurate estimation of the attenuation coefficients in the sinus region.

### Method limitations and applicability across PET-MRI systems

There are some limitations in the proposed methods. In general, the cuboid method is reliant on the accurate segmentation of the MR images as air and soft tissue masks are to define the initial location of the sinus region. The template method is more robust to segmentation errors, but is less sensitive for changes in individual anatomy.

Furthermore, the MRI-CT conversion model does not currently include bone conversion, even though some bone is present in the sinus area. With T1-weighted MRI, bone voxels could not be reliably separated by their MRI intensity (Appendix C). As the largest volume of voxels lay on the 0 HU line, using that as an upper limit minimizes the bias for a random bone voxel. This approximation did not result in large inaccuracies as Table 2 shows.

There are also prerequisites before applying the methods to different PET-MRI systems and MRI sequences. The first is to ensure an accurate segmentation of the probability maps. As the segmentation should be applicable to wide range of MRI sequences, this step is needed to be performed only once, followed by visual quality assurance. For example, we’ve recently assessed segmentation quality with Dixon MR images [39]. When these methods are applied to more challenging applications such as MRI-based radiotherapy (MRI-RT) planning, the geometric accuracy of the used MRI sequence should be also evaluated.

Another prerequisite is the calculation of the model parameters for MRI-CT conversion. As the model is intensity-based, it needs to be adjusted for different MRI contrasts. However, once the parameters for the method are fixed using a set of training subjects, the method can be reliably applied to further subjects outside the training set.

## Conclusions

In conclusion, the methods introduced in this proof-of-concept work provide a basis for further work in improving segmentation-based MRAC and show the benefit of TOF imaging in reducing the error in PET-MRI imaging of the brain. The effect of attenuation modelling in the sinus region to PET quantification in the brain was modest.

## Supplementary Information

**Additional file 1:** Appendix A for detailed description of the methodology for air cavity delineation, Appendix B for details of the methodology used for summing matrix elements efficiently, and Appendix C for full summary of data points used for sinus conversion.

## Data Availability

Although all data cannot be shared because of patient confidentiality, some data generated or used during the study are available from the corresponding author by request. The source codes for the methods presented in this paper can be downloaded free of charge from the GitLab account (https://gitlab.utu.fi/jjlind) of the corresponding author, hosted by University of Turku, or by contacting the corresponding author directly (J. Lindén).

## Abbreviations

AC: Attenuation correction
atMR: Anatomical MR
CT: Computed tomography
CTAC: CT-based attenuation correction
FA: Flip angle
FFE: Fast field echo
HU: Hounsfield unit
MNI: Montreal Neurological Institute
MR: Magnetic resonance
MRF: Markov random field
MRI: Magnetic resonance imaging
MRAC: MR-based attenuation correction
MRI-RT: MRI-based radiotherapy
PET: Positron emission tomography
SPM: Statistical Parametric Mapping software
TE: Echo time
TOF: Time-of-flight
TR: Repetition time
TXAC: Transmission-based attenuation correction
UTE: Ultra-short echo time
VOI: Volume of interest
ZTE: Zero echo time
