# Supplementary material for: Evaluation of three methods for delineation and attenuation estimation of the sinus region in MR-based attenuation correction for brain PET-MR imaging
Source: BMC Med Imaging. 2022 Mar 17;22:48. doi: 10.1186/s12880-022-00770-0 (PMC8928695; doi:10.1186/s12880-022-00770-0)
Supplement: Supplementary file 1 — Additional file 1: Appendix A for detailed description of the methodology for air cavity delineation, Appendix B for details of the methodology used for summing matrix elements efficiently, and Appendix C for full summary of data points used for sinus conversion. [file 12880_2022_770_MOESM1_ESM.docx]

# Supplementary information

## Appendix A – Delineating Air Cavities

Once an initial mask of the air segments inside the head was created, the voxels in each slice of the mask were summed, and these sums were made into a line plot. The first wide peak was chosen by finding the largest local maximum preceding the largest drop in the inside air, assuming that the sinus area is a large air compartment located in the lower region within the head, and above the sinus there are no large compartments of air within the head. This corresponds with the idea that when ascending the slices, the inside air of sinus regions is the last large concentration of air and finding the largest concentration just before the drop would be the sinus area. The image slice containing the peak was then eroded with a disk that has radius of 2 voxels. This way the noise and the voxels that do not anatomically match air cavities were removed, and the remaining areas of the image can be said to be actual air inside the sinus area. For each remaining object, a point corresponding to the center of mass (COM) was calculated.

From each COM point, a recursive region growing algorithm was initiated. First the COM point was added to a new cavity mask. Then each neighboring voxel (three dimensional 6-neighbourhood) of the COM point in the original air and soft tissue mask was inspected. If they had a value of 1 in the mask, and didn’t not yet belong to the cavity mask, they were added to the cavity mask, and then the algorithm was initiated from that neighboring voxel. If the neighbor had a value of 0 in the air and soft tissue mask, or it already belonged to the cavity mask, it was ignored, and next neighbor was inspected. The algorithm would continue to recursively call itself in neighboring voxels until one of the following stopping conditions was met: the neighbor chain is 20 voxels long from the original starting point, or no new neighboring voxels belonging to the air and soft tissue mask were found. This way the sinus cavity will eventually converge even if there is excessive amount of noise in the original air and soft tissue segment image. These cavities are then set as air in the final image.


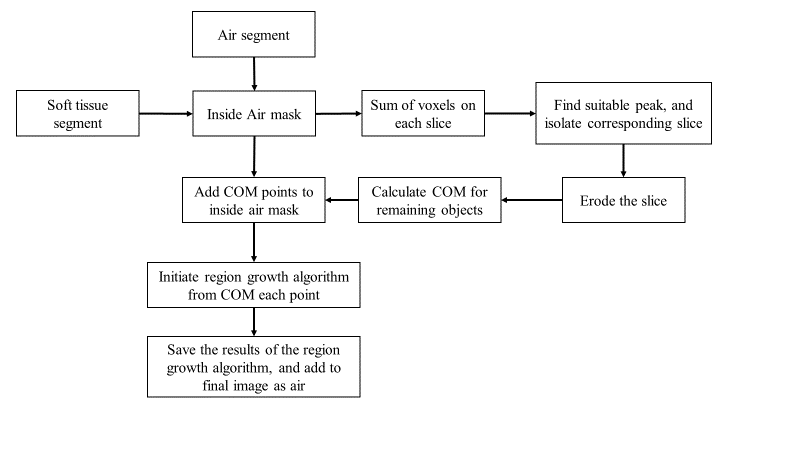


**Supplementary Figure 1.** Air cavity algorithm process flow. The air and soft tissue probability maps are the inputs and the air cavity mask is the output.

## Appendix B – Individual Sinus Cuboid and cuboid sums

The mean position of the center points from the cavity mask process described in Appendix A are used to locate the starting point of the cuboid search. The cuboid search will iteratively make a larger cuboid and vary its position around the starting point. Let us denote the dimensions of an image by $x_{max}, y_{max}, z_{max}\geq1$. We denote a cuboid by 3-dimensional binary matrix ${C=\left( C \right)}_{i,j,k}$ with values 0 except for indices ${i=x_{s},x_{s}+1,\ldots,x}_{e}$, ${j=y_{s},y_{s}+1,\ldots,y}_{e}$ and ${k=z_{s},z_{s}+1,\ldots,z}_{e}$, where ${1\leq x_{s}\leq x_{e}\leq x}_{max}$, ${1\leq y_{s}\leq y_{e}\leq y}_{max}$ and ${1\leq z_{s}\leq z_{e}\leq z}_{max}$. For each image we denote probability value within the cuboid by matrix $\left( B \right)_{i,j,k}$, with same dimensions as $C$. The probability value is now the value of the voxel of the bone segment of the segmented MR image. The goodness criteria for the cuboid is the sum of the probability value of the bone segment image within the cuboid, divided by the square of the length of the cuboid perimeter:

$CG(C)=\frac{\sum_{{i=x}_{s}}^{x_{e}} \sum_{j=y_{s}}^{y_{e}} \sum_{{k=z}_{s}}^{z_{e}} B_{i,j,k}}{\left( \frac{c\left( C \right)}{4} \right)^{2}}$ ,

$$c\left( C \right)=4\left( \left( x_{e}-x_{s}+1 \right)\left( y_{e}-y_{s}+1 \right)\left( z_{e}-z_{s}+1 \right) \right),$$

where $CG$ is the value of the cuboid goodness, $C$ is the cuboid being tested, $c(C)$ is the perimeter of the cuboid $C$, and $b_{i}$ is the bone segment probability value for the voxel $i$.

Having the square in the denominator prevents too large cuboids being chosen since the denominator value will grow rapidly when the perimeter grows. Perimeter also favors more squared cuboids than flat ones since the perimeter to volume ratio is far greater for the latter. For similarly shaped cuboids, growing each edge with the factor of $\sqrt[3]{2}$, will double the volume while only increasing the perimeter by factor of 1.26. The power of two in the denominator also cancels this out somewhat, bringing the denominator value closer to linear. Once all the possible cuboids have been examined, the one with the largest cuboid goodness value is selected. Finally, the cuboids are extended few slices in upwards and forward to make sure it includes the nose and the sinuses of the subject.

The number of cuboids to be inspected is large and the cuboids can contain thousands of voxels, the sum of which needs to be calculated, so a way to reduce the computational complexity was devised. The method is similar to the method previously published in (40). Instead of calculating the sum of bone probability for each cuboid by accessing the bone segment voxels repeatedly a growing sum matrix is created. Every cuboid in a set that shares a chosen corner is inspected in a specific sequence so that each voxel of the bone probability matrix needs to be accessed only once per set of corner-sharing cuboids. The bone probability of the shared corner is set to be the corner of the sum matrix and corresponds to the cuboid of size 1 in all dimensions. Every other value in the sum matrix corresponds to the sum of a cuboid, dimensions of which are defined by spanning a cuboid between the position of the starting corner and the value in question. The values in the sum matrix are generated by always taking a suitable linear combination of the sum matrix values that have been calculated earlier and adding the bone probability value of the voxel in the position of the sum matrix that is being calculated. This reduces the amount of value calls needed to calculate the bone probability sum of a cuboid to maximum of eight calls, whereas directly calculating the bone probability sum of a cuboid can include thousands of value calls to the bone probability segment image. More detailed description of the method can be found below.

At the same time with sum matrix calculation, the perimeters in the denominator are calculated and stored in another matrix. When all cuboids for in a set that share a corner have been assessed the cuboid goodness can be evaluated by dividing the matrices elementwise. If the largest cuboid goodness is larger than the best cuboid goodness of another set of cuboids that share a corner, then the spanning corners of the newly found best cuboid are saved and the assessment continues with the next set of cuboids that share a corner.

To faster calculate all the possible cuboid sums it is important to calculate them in the correct order. Let the set $\Gamma_{C}$ include all the cuboids $C_{1}, \ldots, C_{n}, C_{m}$that fit inside the largest possible sinus region cuboid $C_{m}$. The cuboids can be defined with coordinates of the voxels of two opposite corners of the cuboids $x_{\omega}^{s},x_{\omega}^{e},y_{\omega}^{s},y_{\omega}^{e},z_{\omega}^{s},z_{\omega}^{e}$, where $x,y,z$ are the voxels corresponding to the dimensions in the cartesian coordinate system, $\omega\in\left\{ 1,2,\ldots,n,m \right\}$ and $s,e$ notate the points for the start and end corners of the cuboid respectively. Now for each $\omega$ the following inequalities hold

$$x_{m}^{s}\leq x_{\omega}^{s}\leq x_{\omega}\leq x_{\omega}^{e}\leq x_{m}^{e}$$

$$y_{m}^{s}\leq y_{\omega}^{s}\leq y_{\omega}\leq y_{\omega}^{e}\leq y_{m}^{e}$$

$$z_{m}^{s}\leq z_{\omega}^{s}\leq z_{\omega}\leq z_{\omega}^{e}\leq z_{m}^{e}.$$

Let the three-dimensional matrix $B$ be the bone probability matrix created by the Segment procedure in SPM12. To estimate the goodness of the sinus box, the all the sums $\sigma_{\omega}$ of the elements $b_{i,j,k}\in B$ within cuboids $C_{\omega}$need to be calculated

$$\sigma_{\omega}=\sum_{i=x_{\omega}^{s}}^{x_{\omega}^{e}} \sum_{{j=y}_{\omega}^{s}}^{y_{\omega}^{e}} \sum_{{k=z}_{\omega}^{s}}^{z_{\omega}^{e}} b_{i,j,k}.$$

The calculation of $\sigma_{\omega}$ requires a lot of calls to the matrix $B$ which incurs considerable processing time when the amount and size of the sums $\sigma_{\omega}$ grows. It is evident that many of the sums $\sigma_{\omega}$share a lot of elements $b_{i,j,k}$ so it is possible to reduce the amount of calls to matrix $B$ considerably by summing the elements in $b_{i,j,k}$ a suitable order.

Consider now matrix $M_{p}$ that collects all the subsums $\sigma_{p}$ of the cuboids $C_{p} \in P\subseteq\Gamma_{C}$. The set $P$ is now the set of cuboids $C_{p}$ that share the corner $x_{p}^{s},y_{p}^{s},z_{p}^{s}$ and have different opposite corners. The location of the opposite corner of a cuboid $C_{p}$ will correspond to the location of the sub sum $\sigma_{p}$ in the matrix $M_{p}$. The elements of $M_{p}$ can be calculated iteratively from the elements that have been calculated before. For the ease of notation, a sub matrix $D_{p}$is defined. The matrix $D_{p}$ is the same size as matrix $M_{p}$ and includes the elements of matrix $B$ in a way that $D_{p}\left( 1,1,1 \right)=B(p_{x}{,p}_{y},p_{z})$ where the indices $p$ denote the start points of the cuboids:

$p_{x}=x_{p}^{s}{,p}_{y}=y_{p}^{s},p_{z}=z_{p}^{s}$. Trivially $M_{p}\left( 1,1,1 \right)=B\left( p_{x}{,p}_{y},p_{z} \right)=D_{p}\left( 1,1,1 \right)$. Following recursive equations can be defined for cases along the $i,j,k$ axes:

$$\left\{ \begin{aligned} M_{p}\left( i,1,1 \right)=M_{p}\left( i-1,1,1 \right)+D_{p}\left( i,1,1 \right) \\ M_{p}\left( 1,j,1 \right)=M_{p}\left( 1,j-1,1 \right)+D_{p}\left( 1,j,1 \right) \\ M_{p}\left( 1,1,k \right)=M_{p}\left( 1,1,k-1 \right)+D_{p}\left( 1,1,k \right). \end{aligned} \right.$$

Following equations can be defined for cases where one of the coordinates $i,j,k$ is 1.

$$\left\{ \begin{aligned} M_{p}\left( i,j,1 \right)=M_{p}\left( i-1,j,1 \right)+M_{p}\left( i,j-1,1 \right)-M_{p}\left( i-1,j-1,1 \right)+D_{p}\left( i,j,1 \right) \\ M_{p}\left( i,1,k \right)=M_{p}\left( i-1,1,k \right)+M_{p}\left( i,1,k-1 \right)-M_{p}\left( i-1,1,k-1 \right)+D_{p}\left( i,1,k \right) \\ M_{p}\left( 1,j,k \right)=M_{p}\left( 1,j-1,k \right)+M_{p}\left( 1,j,k-1 \right)-M_{p}\left( 1,j-1,k-1 \right)+D_{p}\left( 1,j,k \right). \end{aligned} \right.$$

And finally, the general recursion equation to situations where all coordinates are greater than 1.

$M_{p}\left( i,j,k \right)=M_{p}\left( i-1,j,k \right)+M_{p}\left( i,j-1,k \right)+M_{p}\left( i,j,k-1 \right)$

$$-M_{p}\left( i-1,j-1,k \right)-M_{p}\left( i-1,j,k-1 \right)-M_{p}\left( i,j-1,k-1 \right)$$

$$+M_{p}\left( i-1,j-1,k-1 \right)+D_{p}\left( i,j,k \right).$$

In order to calculate the next element in $M_{p}$ only up to seven calls needs to be made to matrix $M_{p}$ and one to matrix $D_{p}$ compared to potentially thousands calls to the matrix $B$ in the earlier sum formula for $\sigma_{\omega}$. The one- and two-dimensional recursion equations written earlier can be reached from the last one by setting any combination of $i,j,k$ as ones, and then removing the terms where any of the indices goes to zero from the equations.

Consider now another set of cuboids, $C_{q}$, which includes the set of cuboids that share the corner $x_{q}^{s},y_{q}^{s},z_{q}^{s}$ in a way that the indices of the start corner are the same or larger for the $C_{q}$ than for the cuboids $C_{p}$

$$p_{x}\leq q_{x}{, p}_{y}\leq q_{y}, p_{z}\leq q_{y}.$$

If $p_{x}=q_{x}{,p}_{y}=q_{y},p_{z}=q_{y}$then trivially $M_{q}=M_{p}$ so it can be assumed that at least one of the inequalities is strict. Recursion equations can be written to calculate the elements of $M_{q}$ from the elements of $M_{p}$. If only one of the indices for starting corner is different the equation is as follows:

$$M_{q}\left( i,j,k \right)=M_{p}\left( i+t,j,k \right)-M_{p}\left( t,j,k \right),$$

if the different index is on x-axis. The index shift number $t=q_{x}-p_{x}$. Similar equations can be written for the recursive relations in y and z-axes.

If two of the indices are different, then the equation becomes:

$$M_{q}\left( i,j,k \right)=M_{p}\left( i+t,j+u,k \right)-M_{p}\left( i+t,u,k \right)-M_{p}\left( t,j+u,k \right)+M_{p}\left( t,u,k \right),$$

where the index shift number $t=q_{y}-p_{y}$. Again, similar equations can be written for situations where other index combinations occur.

Finally, if all the indices are different the general equation is as follows:

$$M_{q}\left( i,j,k \right)=M_{p}\left( i+t,j+u,k+v \right)$$

$$-M_{p}\left( i+t,j+u,v \right)-M_{p}\left( i+t,u,k+v \right)-M_{p}\left( t,j+u,k+v \right)$$

$$+M_{p}\left( i+t,u,v \right)+M_{p}\left( t,j+u,v \right)+M_{p}\left( t,u,k+v \right)-M_{p}\left( t,u,v \right),$$

where the index shift number is $v=q_{z}-p_{z}$. Now the one- and two-dimensional recursion equations written earlier can be reached from the last one by setting any combination of $t,u,v$ as zeroes, and then eliminating the terms where any of the indices goes to zero. If the first subset $C_{p}$contains the largest possible cuboid $C_{m}$then the corresponding subsum matrix $M_{m}$allows the easy calculation of any other sub sum matrix $M_{q}$. However, for the general case there are as many calls to memory as when calculating $M_{p}$ from $D_{p}$, so the computational gains can be small. But with parallel processing gains can be obtained, since the latter method calls only already calculated matrix, so any element can be calculated in any desired order, unlike the earlier method that calculates the elements in target matrix in some determined order using the earlier calculated elements.

## Appendix C – Summary of Sinus Conversion data points


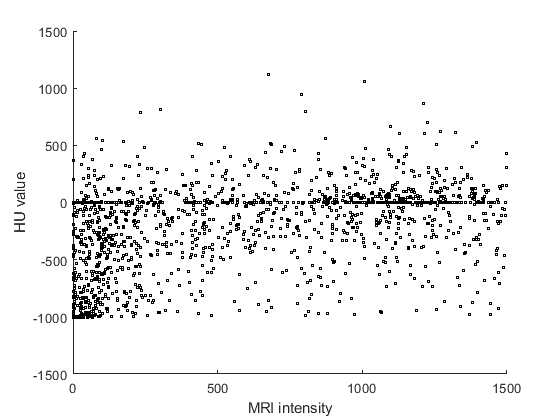


**Supplementary Figure 2.** Summary of the data points of all MRI-CT sinus conversion samples. The voxels that are considered as bone based on their HU-values cannot be identified using their MRI intensity alone.
